# RNA methylations in hepatic fibrosis, a gradually emerging new treatment strategy

**DOI:** 10.1186/s13578-023-01066-8

**Published:** 2023-07-07

**Authors:** Chenglong Cheng, Yajie Wu, Xin Wang, Qiuyun Xue, Yurong Huang, Faxue Liao, Xiao Wang, Qiangjun Duan, Chenggui Miao

**Affiliations:** 1 Department of Pharmacology, School of Integrated Chinese and Western Medicine, Anhui University of Chinese Medicine, Hefei, China; 2 Department of Orthopaedics, The First Affiliated Hospital, Anhui Medical University, Hefei, China; 3 Anhui Public Health Clinical Center, Hefei, China; 4 Department of Clinical Nursing, School of Nursing, Anhui University of Chinese Medicine, Hefei, China; 5 Department of Experimental (Practical Training) Teaching Center, School of Integrated Chinese and Western Medicine, Anhui University of Chinese Medicine, Hefei, China; 6 Institute of Rheumatism, Anhui University of Chinese Medicine, Hefei, China

**Keywords:** Epigenetics, Hepatic fibrosis, RNA methylation, N6-methyladenosine, Hepatic stellate cells

## Abstract

**Background:**

Hepatic fibrosis (HF) is a pathological process caused by excessive accumulation of extracellular matrix caused by a series of causes, leading to the formation of fiber scar. RNA methylation is a newly discovered epigenetic modification that exists widely in eukaryotes and prokaryotes and plays a crucial role in the pathogenesis of many diseases.

**Results:**

The occurrence and development of HF are regulated by many factors, including excessive deposition of extracellular matrix, activation of hepatic stellate cells, inflammation, and oxidative stress. RNA methylations of different species have become a crucial regulatory mode of transcript expression, And participate in the pathogenesis of tumors, nervous system diseases, autoimmune diseases, and other diseases. In addition, there are five common types of RNA methylation, but only m6A plays a crucial regulatory role in HF. The pathophysiological regulation of m6A on HF is achieved by the combination of the methylated transferase, demethylated enzyme, and methylated reading protein.

**Conclusions:**

RNA methylated methyltransferase, demethylase, and reading protein extensively affect the pathological mechanism of HF, which may be a new therapeutic and diagnostic target, representing a new class of therapeutic strategies.

## Background

Hepatic fibrosis (HF) is a pathological process in which various chronic liver injuries cause excessive accumulation of extracellular matrix (ECM), leading to continuous repair of injury [1]. The perisinusoidal space, also known as the Disse space, is a narrow space about 0.4 μm wide between hepatocytes and endothelial cells in the blood sinuses. Abnormal activation of hepatic stellate cells (HSCs) in the Disse space and accumulation of ECM and other components are crucial events in the development of HF [2–4]. The involvement of ECM and other components is a key event in trauma healing and tissue repair (tissue damage, infection, inflammation, etc.) [5–7]. The pathogenesis of HF is influenced by multiple factors. Any factor that can lead to chronic damage of liver tissue can induce the development of HF. For example, inborn metabolic defects, alcohol abuse, viral infections, parasitic infections, and autoimmune liver disease [8–12]. In addition, an elevated body mass index further increases the risk of HF due to non-alcoholic fatty liver disease (NAFLD) and nonalcoholic steatohepatitis (NASH) [13–15]. Early-stage HF without proper management and treatment will further progress to cirrhosis and hepatocellular carcinoma with serious consequences [16].

Post-transcriptional regulation is the further modification and processing of eukaryotic gene transcription products, including various processes such as processing, translocation, translation, and degradation throughout the life cycle of RNA molecules [17, 18]. RNA post-transcriptional regulation has more significant advantages than transcriptional regulation in regulating disease onset and progression [19]. RNA methylations belong to a type of RNA post-transcriptional regulation, which mediates almost all aspects of RNA processing, including RNA splicing, stability, degradation, and translation [20–25].

N6-methyladenosine (m6A) modifications are among the most abundant modifications in eukaryotic mRNAs. Methylation transferases and demethylases are crucial enzymes that jointly regulate m6A modification [26]. M6A's role in the pathogenesis of HF has been gradually elucidated, which will provide new perspectives for diagnostic and therapeutic studies of HF.

The reversible epigenetic modification 5-methylcytosine (m5C) has also been extensively studied, especially in the development of tumors. M5C is found in a variety of representative biological mRNAs, rRNAs, and tRNAs. M5C affects the function of modified RNA molecules and is essential for many biological activities, including control of transcription, protein interactions, and RNA stability [27].

In addition to the common m6A and m5C modifications, N1-methyladenosine (m1A), N6,2-O-dimethyladenosine (m6Am), and 7-methylguanosine (m7G) are also RNA methylations modifications. M1A is a post-transcriptional modification with a high abundance of eukaryotic rRNAs and tRNAs. Unlike m6A, m6Am is mainly located on the first base after the 5' cap of eukaryotic mRNAs. M7G modifications act by affecting the metabolism of various RNA molecules, including mRNAs, tRNAs, microRNAs, and rRNAs. However, the relationship between RNA methylation modifications other than m6A and HF has not been reported.

Given that RNA methylations have become a new focus of research, their function is gradually being discovered. The present work focuses on the role and mechanism of RNA methylation in HF and reveals its molecular features and biological functions.

## Mechanisms of hepatic fibrosis

**Fig. 1:**
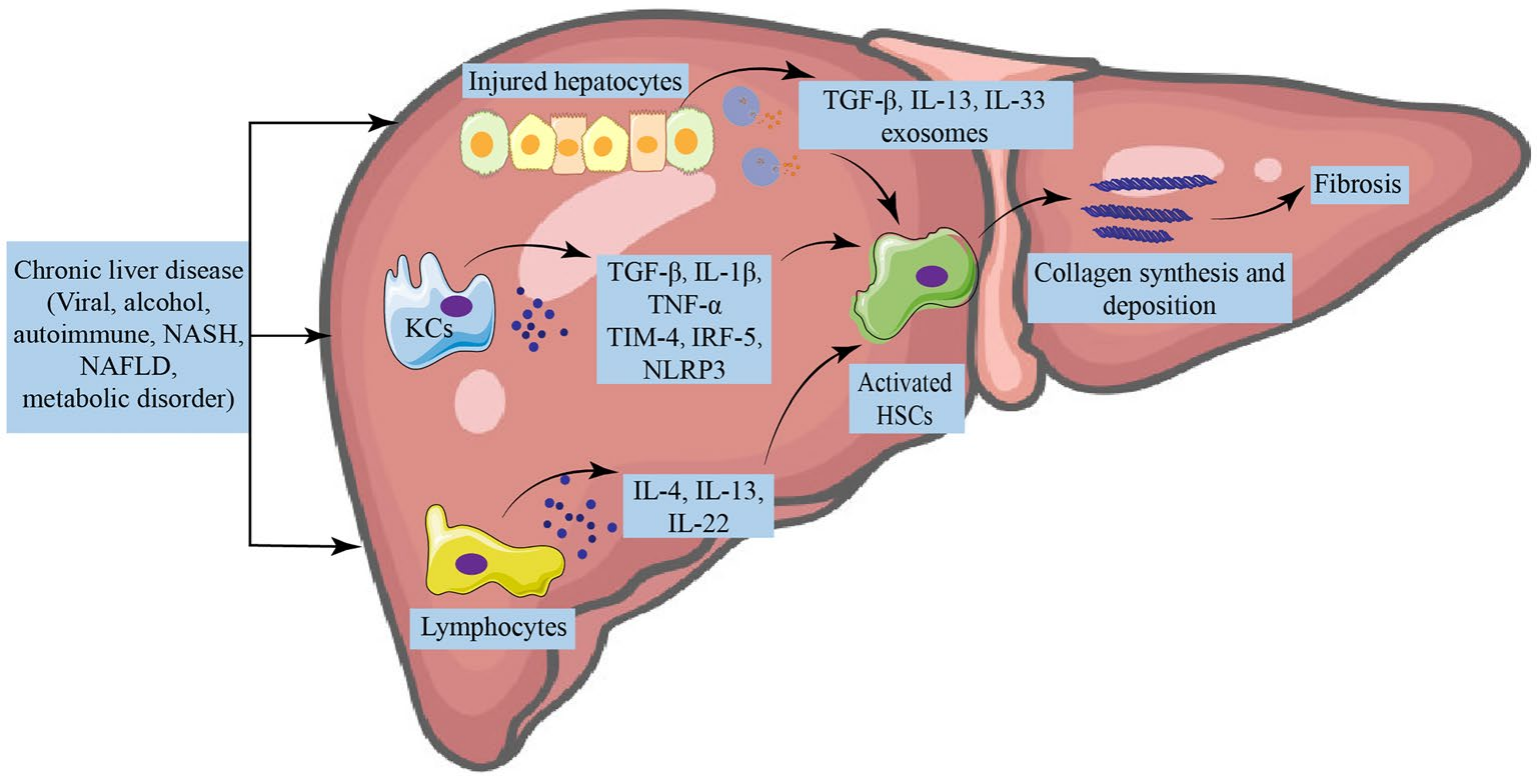
Mechanisms of hepatic fibrosis. Chronic liver injury mediated by different risk factors activates several parenchymal and non-parenchymal cells and promotes hepatic inflammation by producing many inflammatory mediators. Extreme inflammation drives the activation of hepatic stellate cells, which are then transformed into the proliferative and extracellular matrix, giving rise to myofibroblasts, leading to fibrosis and liver dysfunction. *NAFLD* non-alcoholic fatty liver disease, *NASH* nonalcoholic steatohepatitis, *KCs* kupffer cells, *TGF-β* transforming growth factor beta, *TNF-α* tumor necrosis factor-alpha, *TIM-4* T-cell immunoglobulin and mucin-4, *IRF-5* interferon regulatory factor-5, *NLRP3* NLR family pyrin structural domain containing 3, *HSCs* hepatic stellate cells

HF is a dynamic process that continuously responds to wound repair. It is not an independent disease, and any liver injury is often accompanied by the occurrence of HF. There are many clinical causes of HF, such as NAFLD, NASH, autoimmune hepatitis, viral hepatitis, etc. In addition, the mechanisms of HF are diverse and complex, including inflammation, oxidative stress, HSCs activation, and ECM over-deposition (Fig. 1).

## Imbalanced of ECM expression induces hepatic fibrosis

Under normal physiological conditions, the dynamic balance of ECM in the liver depends on ECM synthesis and matrix metalloproteinases (MMPs)-mediated degradation of ECM. The main physiological function of MMPs is the direct degradation of ECM (collagen, laminin, and elastin) [28]. ECM, MMPs, and tissue inhibitors of metalloproteinases (TIMPs) that inactivate MMPs are simultaneously synthesized and secreted by HSCs. However, sustained expression of TIMPs in the damaged liver inhibits the activity of MMPs, which further prevents the degradation of collagen fibers [29].

In the carbon tetrachloride (CCL4)-induced HF model in mice, collagen I expression is significantly upregulated. Isorhamnetin can not only significantly inhibit HSCs activation, but also inhibit ECM formation and autophagy by down-regulating TGF-β1-activated Smad3 and p38MAPK signaling pathways [30]. CHR-HPBCD and CHR-RAMEB are two complexes of leucovorin (CHR), which inhibit collagen deposition and reduce the expression of inflammatory factors. Mechanistically, CHR-HPBCD and CHR-RAMEB downregulate the TGF-β1/Smad signaling pathway and NF-κB-mediated inflammatory pathway and regulate anti-HF-related miRNA expression, which in turn exerts anti-inflammatory and anti-fibrotic effects [31]. The development of HF is influenced by the massive production and activation of myofibroblasts (MFB). Hepatocytes are an important component of MFB and can be converted to MFB through epithelial-mesenchymal transition (EMT) during HF. Curcumin effectively regulates PPARα and oxidative stress to promote autophagy activation, which effectively reduces the occurrence of EMT in hepatocytes and inhibits the accumulation of ECM [32].

## HSCs activation is the central link of hepatic fibrosis formation

HSCs are located in the Disse space canal and account for 15% of the liver's cells and about 33% of the non-parenchymal cells. Under normal conditions, resting HSCs are ovoid or irregular in shape. A large amount of vitamin A and primary forms of lipid droplets, as well as retinoid derivatives, are present intracellularly in HSCs [33, 34]. However, when the liver is damaged by inflammation or mechanical stimulation, the resting HSCs are activated, and their phenotype undergoes a shift from the resting to the activated form, which is a central step in the development of HF [35].

Activated HSCs are an important source of MFB, and other cells can also differentiate into MFB [36]. The conversion of HSCs into phenotypic MFB is influenced by many factors, such as viral hepatitis, alcoholic liver disease, and autoimmune hepatitis. The main function of MFB is to secrete ECM (including smooth muscle actin, collagen, fibronectin, laminin, and proteoglycans), which promotes damaged liver healing [37]. However, when risk factors persist, HSCs continue to activate and convert to MFB. This leads to a massive accumulation of collagen fibril-based ECM and disrupts the homeostasis of the Disse. Further, a sustained increase in insoluble fibers and structural alteration events in the liver occurs, ultimately leading to continued exacerbation of HF and malignant transformation [38]. Therefore, inhibiting static HSCs activation and promoting activated HSCs apoptosis may be a new therapeutic direction for HF.

Transforming growth factor β (TGF-β) and platelet-derived growth factor (PDGF) are the two most critical factors for HSCs activation. The prominent role of TGF-β is to promote the formation of collagen and matrix while inhibiting its degradation [39]. In the mitogenic pathway, PDGF is the most potent factor in promoting HSCs signaling, and in particular, β-PDGFR has the strongest proliferative effect on HSCs [40]. PDGF receptors and ligands are up-regulated in most HF models and human HF patients [41, 42]. They promote collagen production and deposition and directly cause HSCs proliferation and conversion to MFB to synthesize and secrete large amounts of ECM [43]. Therefore, blocking PDGF signaling inhibits HSCs proliferation and ameliorates HF.

Kupffer cells (KCs) are specialized macrophages located in the inner wall of hepatic sinusoidal cells. KCs are plastic and are involved in HSCs activation and fibrosis formation [44]. KCs can differentiate into M1 or M2 phenotypes. M1 KCs can not only secrete inflammatory factors such as IL-1β and TNF-α, but also are closely related to promoting liver inflammatory response. M2 KCs secrete IL-10, TGF-β, and PDGF and are associated with the inhibition of inflammatory responses and tissue repair [45, 46]. In the early stages of pathology, M2 KCs create an ant-inflammatory environment and promote tissue repair by ECM remodeling and fibroblast recruitment, but do not promote fibrosis. However, when lesions persist, M2 KCs gain a role in pro-fibrosis through the excretion of large amounts of TGF-β as well as galactose lectin-3, causing induction of fibrosis [47, 48].

The functions of liver resident lymphocytes include immune surveillance and maintenance of hepatic homeostasis. Yet, the liver's permanent hepatic lymphocytes are protective and pathogenic leading to hepatitis, fibrosis, and cirrhosis under pathological conditions. NKT cells are enriched in hepatic lymphocytes and generate high levels of fibrogenic cellular factors (IL-4, IL-13) that promote the activation of HSCs [48, 49]. Leptin is derived from the 16 kDa adipocytokine product of the obesity gene and exhibits a variety of pro-fibrotic properties. In addition, the effects of appetite, insulin secretion, and glucose metabolism are regulated by leptin [50]. Leptin induces oxidative stress in HSCs and stimulates TIMP-l expression and inhibits MMP-1 expression and activity, which in turn promotes fibril formation in HSCs [51, 52].

Angiotensin II (AngII) is a vasoconstrictor peptide of the renin-angiotensin system (RAS). AngII leads to increased intrahepatic resistance and induces TGF-β1 to promote ECM deposition through its vasoconstrictive effects, which in turn enhance proinflammatory mediators and HF [53]. MicroRNA-21 is significantly up-regulated in patients with HF. In primary HSCs, AngII upregulates microRNA-21 expression by targeting Smad7 and Spry1, whereas Ang (1–7) inhibits HF and AngII-induced microRNA-21 expression [54].

## Inflammation and hepatic fibrosis

Inflammatory response induced by liver injury is a typical event of progressive fibrosis. The normal inflammatory response is conducive to the healing of the liver injury site. However, when the liver is subjected to continuous inflammatory stimulation, it can lead to HF and irreversible liver damage such as cirrhosis or hepatocellular carcinoma.

In liver injury, interleukins are produced in large quantities by a variety of cell types and play pro-inflammatory (IL-6, IL-13, IL-17, and IL-33) and anti-inflammatory (IL-10) functions in liver cells [55]. IL-6 is a representative key cytokine in liver disease and has a pro-inflammatory effect. IL-6 can directly induce the transformation of HSCs into myofibroblast-like cells and promote the occurrence of HF [56]. IL-13 is an immunomodulatory cytokine secreted primarily by Th2 cells. IL-13 has been proven to be the primary cytokine causing fibrosis, which can bind to IL-13 receptor alpha1 (IL-13Rα1) to induce fibrosis [57]. The expression of IL-17 in liver tissue of HF patients is significantly up-regulated, and the high expression of IL-17 promotes fibrosis markers and IL-6 secretion [58] In addition, the activation of the pro-fibrotic TGF-β signaling pathway is driven by several collaborative mechanisms, in which the pro-inflammatory cytokine IL-17A plays a prominent role [59]. There is evidence that microbial-driven intestinal fibrosis may be mediated by inducing the fibrosis-promoting action of IL-33 receptor ST2 on epithelial cells. The intestinal dysbiosis will further lead to HF [60].

IL-22, a member of the IL-10 cytokine family, acts as a hepatocyte survival factor and binds to receptors IL-22R1 and IL-10R2 to play a protective role in a variety of liver diseases, such as hepatitis, HF, and hepatocellular carcinoma [61]. IL-22 can improve liver oxidative stress and alcoholic fatty liver by activating liver signal transducer and transcription activator 3, and effectively alleviate liver injury caused by alcohol and toxic substances [62]. In addition, overexpression of IL-22 can reduce HF by decreasing HSCs activation and down-regulating inflammatory cytokine levels [63]. In conclusion, the liver conservation function and liver regeneration promotion of IL-22 indicate the therapeutic potential of IL-22 in the treatment of human liver diseases.

The process of inflammation triggering HF is complicated, and the effect of inflammation on fibrosis still needs to be explored and studied continuously. Further understanding of the role of inflammatory cells and cytokines in HF is helpful to elucidate the pathogenesis of HF and develop new clinical drugs.

## Oxidative stress and hepatic fibrosis

More and more evidence shows that oxidative stress plays an important role in the occurrence and development of HF. Oxidative stress is involved in the process of HF caused by various diseases. Oxidative stress refers to a state of excessive production of reactive oxygen species (ROS) in the body or cells or weakening of antioxidant function in the body, which seriously disrupts the balance between the two, leading to inflammation and lipid peroxidation, resulting in tissue and cell damage.

In the damaged liver, activated KCs, activated HSCs, and neutrophils can activate the oxidative stress system to produce ROS and interfere with the normal function of liver-specific cells. Therefore, restoring the balance between oxidation and antioxidant systems in vivo can improve HF [64, 65]. Maresin-1 can improve HF by promoting hepatocyte proliferation and reducing oxidative stress and inflammation [64].

Nuclear factor erythroid2-related factor 2 (Nrf2) is an important antioxidant stress factor in vivo and the central link of the liver antioxidant stress system. Nrf2 activation can enhance endogenous antioxidant systems and resist oxidative stress systems imbalance in vivo [66]. Gardeniae Fructus significantly attenuates TGFβ1-induced ECM accumulation in LX-2 cells through the AMPK/SIRT1 pathway and Nrf2, thereby alleviating HF [67]. Bone marrow mesenchymal stem cells up-regulate the expression of Nrf2 and HO-1 in the liver tissue of CCl4-poisoned rats, suggesting that they inhibit oxidative stress, inflammatory response and liver fibrosis by activating Nrf2/HO-1 signaling pathway [68]. In conclusion, improving the body's antioxidant stress ability is expected to be a feasible strategy to treat HF.

## RNA methylations

**Fig. 2:**
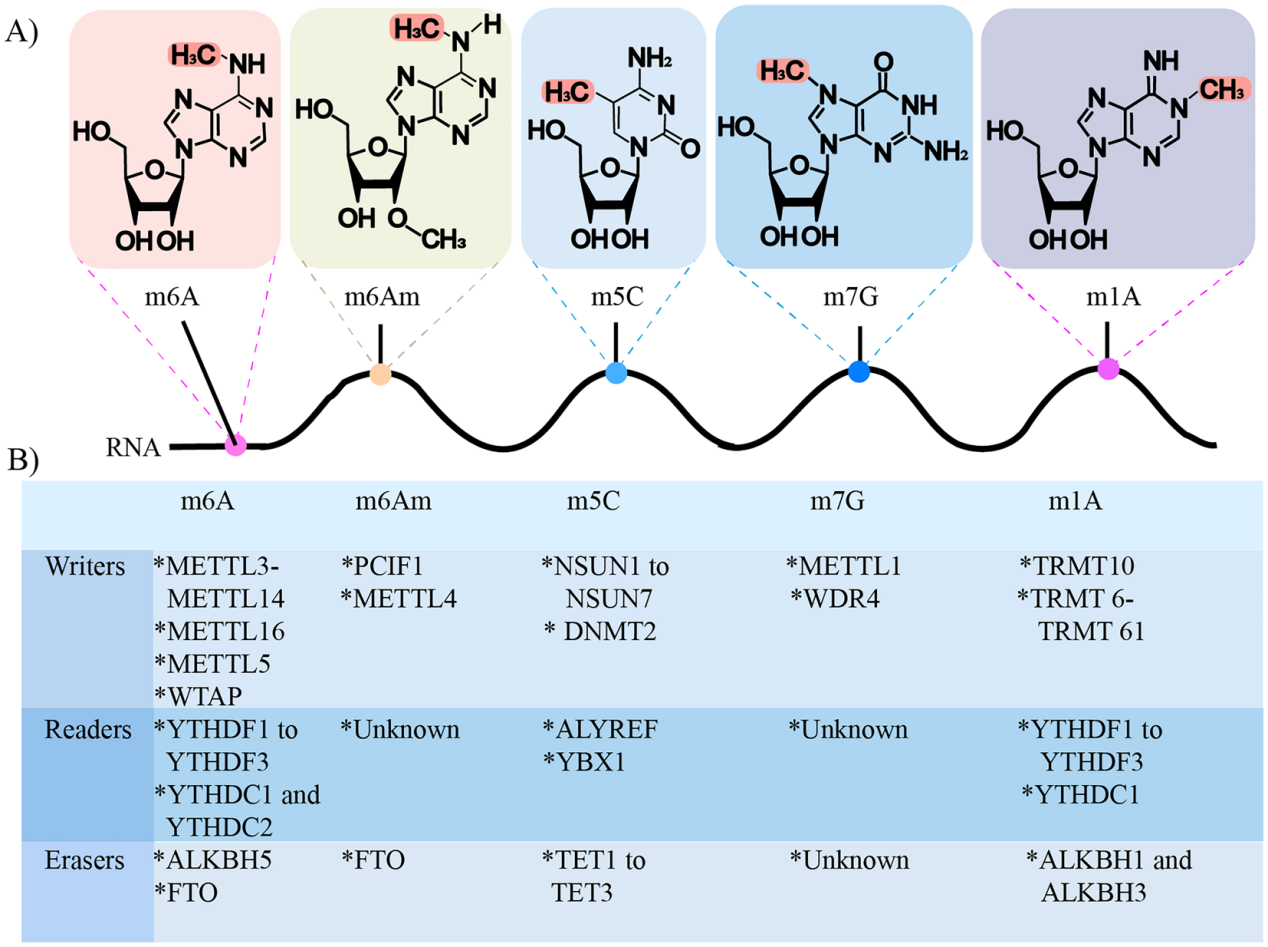
Molecular structures of RNA methylation. **A** Molecular structures of the five methylation-modified RNAs. The RNA methylation involved includes (m6A, m6Am, m5C, m1A, m7G); **B** the five RNA methylation corresponding modification enzymes, including (methylesterase, methylation recognition protein, and demethylase)

RNA methylations of different species have become a crucial regulatory mode of transcript expression. So far, RNA methylation modifications have been found in mammals, including m6A, m6Am, m5C, m7G, m1A, etc. They are catalyzed by RNA methyltransferase (Writers), demethylated by demethylase (Erasers), and read by methylated binding proteins (Readers). RNA methylation mediates almost all aspects of RNA processing, including splicing, nucleation, stability, degradation, and translation of RNA. Therefore, RNA methylation is closely related to tumors, nervous system disorders, autoimmune diseases, and other diseases. In addition, the function of RNA methylation gradually appears in HF, and m6A in particular plays a crucial role in the occurrence and development of HF (Fig. 2).

## M6A modification

Desrosiers et al. [69] first identified the m6A modification in rat mRNAs in 1974. M6A modification is a reversible process commonly found in yeast, plants, bacteria, humans, and other mammalian mRNAs [70]. The m6A modification is highly enriched in the non-coding region, near the stop codon of mRNA. M6A affects biological processes such as RNA folding, stability, and degradation, which make it involved in splicing, translation, export, and decay [71].

The modification of m6A in mammalian cells is catalyzed by the methyltransferase complex, which consists of methyltransferase like 3 (METTL3), methyltransferase like 14 (METTL14), and the wilms tumor 1 associated protein (WTAP) [72]. WTAP plays a crucial role in embryonic development and interacts with METTL3 and METTL14 to form heterodimers that participate in m6A RNA methylation [73].

Fat mass and obesity-associated protein (FTO) and human AlkB homolog 5 (ALKBH5) are the main demethylases for m6A modification. FTO is associated with obesity and is a member of the Alkb protein family. FTO differs from other proteins in the Alkb family in that the FTO protein ALKBH5 is another demethylating enzyme and directly removes m6A modifications from RNA. In addition, m6A modification regulates mRNA splicing, export, translation, and degradation, which is exerted by changing RNA structure or recruiting m6A modification recognition proteins. Currently, common recognition proteins are YTHDF1, YTHDF2, and YTHDF3.

## M6Am modification

Usually, the 5' end of mRNA carries the m7GPPPN structure, and the first or second nucleotide of this structure can be methylated at the 2'-hydroxyl group. When the first nucleotide of the m7G hat structure is 2′-O-methyladenosine (Am), it can be further methylated at the N6 position to become m6Am [74]. Unlike m6A, m6Am is precisely located adjacent to the cap structure of mRNA at the first transcribed nucleotide in eukaryotes [75].

PCIF1, a factor that interacts with the serine-5-phosphorylated carboxy-terminal structural domain of RNA polymerase II, is the m6Am-modifying enzyme of mRNA. PCIF1 specifically recognizes the 5' cap on mRNA and exerts m6Am methyltransferase activity, but its regulatory mechanism has not been clarified [76].

## M5C modification

M5C is found in mRNAs, rRNAs, and tRNAs. It is characterized by affecting the functions of modified RNA molecules, including transcription, protein interactions, and RNA stability.

The currently identified coders of m5C genes include the NSUN family (NSUN2, NSUN6) and the DNMT family (TRDMT1, TRM4B) [77]. NSUN2 is one of the m5C methyltransferases and is mainly responsible for tRNA and mRNA methylation. NSUN2 Proteins utilize two active sites on the active site cysteine catalytic site, consisting of the 5th carbon atom, to perform halophile action on the methyl form of SAM and complete methylation. Unlike NSUN2, methylation of TRDMT1 uses cysteines only at a single site.

The readers of m5C include ALYREF, Y-box binding protein 1 (YBX1), and the DNA repair protein RAD52 homolog (RAD52) [78]. ALYREF mainly regulates the output of pass-through mRNAs, a function that depends on the specific binding of K171 (lysine at position 171) to m5C-modified mRNAs. RAD52 is characterized by a high affinity for hybrid strands containing m5C-modified RNA and DNA, which determines that RAD52 is an m5C reader for DNA damage sites.

## M7G modification

The m7G modification is a self-positively charged RNA methylation modification in which the methyl group binds to the 7th nitrogen atom of RNA guanine, catalyzed by methylation transferase. M7G is present in most eukaryotic and viral mRNAs and is mainly enriched in the start codon region of mRNA [79]. M7G cap formation occurs during substrate mRNA transcription and is catalyzed by the recruitment of the RNA polymerase II enzyme to catalyze it. This structure affects many biological processes, including package transcription, splicing, nuclear export of mRNA, translation, and mRNA stability [80].

METTL1/WDR4 mediates m7G modification of the 5'-UTR region of mRNAs. In mammals, tRNAs are indirectly affected by the METTL1/WDR4 complex-regulated m7G modification, which is essential for normal biological growth, and this pathway also regulates the mRNA translation process and ribosome biosynthesis [81].

## M1A modification

M1A, first identified in tRNA in 1966, is an important post-transcriptional RNA modification that places a methyl ester at the N1 bit of adenosine [82]. M1A methylation occurs mainly in rRNAs and tRNAs, is involved in the maintenance of RNA tertiary structure, and affects protein translation efficiency. Similar to m6A modification, m1A is also a dynamic and reversible RNA modification mediated by RNA methylation modifying proteins.

TRMT6-TRMT61A is a methyltransferase complex that binds to m1A modification at human tRNA 58th (m1A58). During retroviral infection, m1A58 acts as a reverse transcriptase termination site and prevents DNA synthesis [83]. ALKBH1 mediates the demethylation of m1A in tRNA and is a tRNA demethylase. ALKBH1 catalyzes the demethylation of target tRNAs, leading to weakened translation initiation and reduced use of tRNAs in protein synthesis. This process is dynamic and affects translation by regulating the availability of glucose [84].

## Biological functions of RNA methylations

### RNA methylations in tumors

Mitochondria are essential for tumorigenesis. Mitochondria are energy-producing organelles in cells, involved in biosynthesis and signaling. Metabolic plasticity confers the ability of tumors to survive in negative states (e.g., hypoxia, starvation), especially in proliferation-independent processes (e.g., the spread of primary tumors) [85].

M5C and its derivative 5-formyl cytosine can drive the translation of mitochondrial mRNA to promote metastasis. Translation of the subunits of the oxidative phosphorylation complex encoded by the mitochondrial genome is dependent on the formation of m5C modifications at the mitochondrial tRNAMet34 position. Altered mitochondrial function and enhanced glycolysis in m5c-deficient human oral cancer cells in vivo do not affect the cellular activity or primary tumor growth. However, when m5C is absent in tumor mitochondria, tumors no longer metastasize efficiently, and metabolic plasticity is severely compromised [86, 87]. Therefore, RNA modifications at mitochondria-specific sites may serve as drug targets against tumor metastasis.

Su et al. [88] first identified METTL16 in the nucleus as a "writer" of m6A modifications involved in mRNA catalysis. In the cytoplasm, METTL16 promotes the assembly of 80 S ribosomes by directly binding to eIF3a/b and rRNAs, thereby promoting protein translation efficiency and tumor development. This suggests that targeting METTL16 is expected to be a potential new strategy for tumor therapy.

### RNA methylations in the nervous system

M6A modifications play a key role in neurophysiological and pathological mechanisms including neurogenesis, the growth of axons, and the plasticity of synapses. Aberrant m6A modifications lead to acute, and chronic central nervous system (CNS) injuries, brain cancer, and neuropsychiatric disorders [89].

Blocking m6A by knocking down METTL4 caused a prolongation of the cell cycle in radial glial cells. Similarly, in the case of METTL3 knockdown, it triggers a reduction of m6A, which prolongs the radial glial cell cycle and role maintenance. In addition, m6A modification signals also regulate cortical neurogenesis in human forebrain tissue [90]. In the adult mouse hippocampal nerve, m6A facilitates protein translation of target transcripts through its binding protein YTHDF1, thereby promoting learning and memory. YTHDF1 deletion resulted in learning and memory deficits and impaired synaptic transmission in mice, which could be rescued by YTHDF1 re-expression. This suggests that YTHDF1 promotes the translation of m6A methylated neuronal mRNA in response to neuronal stimulation, a process that contributes to learning and memory [91].

### RNA methylations in metabolic diseases

Metabolic diseases are diseases caused by disorders in the metabolism of proteins, fats, and carbohydrates, including diabetes, gout, and osteoporosis. Islet cell biology is critically regulated for glucose homeostasis. Sequencing of m6a in human type 2 diabetic islets revealed several aberrantly methylated transcripts, including those involved in cell cycle progression, insulin secretion, and the insulin/IGF1-AKT-PDX1 pathway. M6A is also involved in the regulation of β-cell biology. m6A levels are down-regulated in EndoC-βH1, leading to cell cycle arrest, and AKT phosphorylation and PDX1 protein levels are suppressed by downregulating insulin secretion [92].

Defective METTL3 function leads to impaired bone formation, insufficient osteogenic differentiation potential, and increased bone marrow adiposity. The PTH (parathyroid hormone)/PTH1r (parathyroid hormone receptor-1) signaling axis is a downstream pathway of m6A regulation in bone marrow mesenchymal stem cells. Knockdown of METTL3 reduces the translation efficiency of PTH1r and disrupts the PTH-induced osteogenic and adipogenic responses in vivo. This fully explains that the upregulation of METTL3 in bone marrow MSCs alleviates osteoporosis caused by estrogen deficiency in mice [93]. Regulation of lipid metabolism through YTHDF2 and PPARα binding is achieved by affecting the stability of mRNA. Knockdown of METTL3 inhibits m6A methylation, resulting in decreased m6A abundance and increased mRNA expression in PPARα, thereby reducing lipid accumulation in cells in vitro [94].

### The RNA methylation of other diseases

In addition to the biological functions of RNA methylation described above, m6A plays a crucial role in regulating other biological processes. For example, m6A affects the splicing processing of miRNA precursors. The deletion of METTL3 reduces the binding of the binding protein DGCR8 to pri-miRNA and leads to the accumulation of pri-miRNA and the reduction of mature miRNA [95].

Many studies in recent years have shown that abnormal m6A methylation is associated with hematopoiesis, heart failure, the respiratory system, reproductive regulation, autoimmune diseases, and growth and development. METTL14 and m6A modifications play a key role in hematopoiesis. The mechanism is through the SPI1-METTL14-MYB/MYC signaling axis of myelopoiesis and leukemogenesis [96]. YTHDF1 is an evolutionarily positively selected high-altitude adaptor gene that is amplified in various cancers, including non-small cell lung cancer (NSCLC). Knockdown of YTHDF1 can regulate the translation efficiency of CDK2, CDK4, and cyclin D1 through the Keap1-Nrf2-AKR1C1 axis, thus exerting an inhibitory effect on NSCLC cell proliferation [97]. YTHDF1 may be a new target for the treatment of respiratory cancers.

### The roles of RNA methylation in hepatic fibrosis

**Fig. 3:**
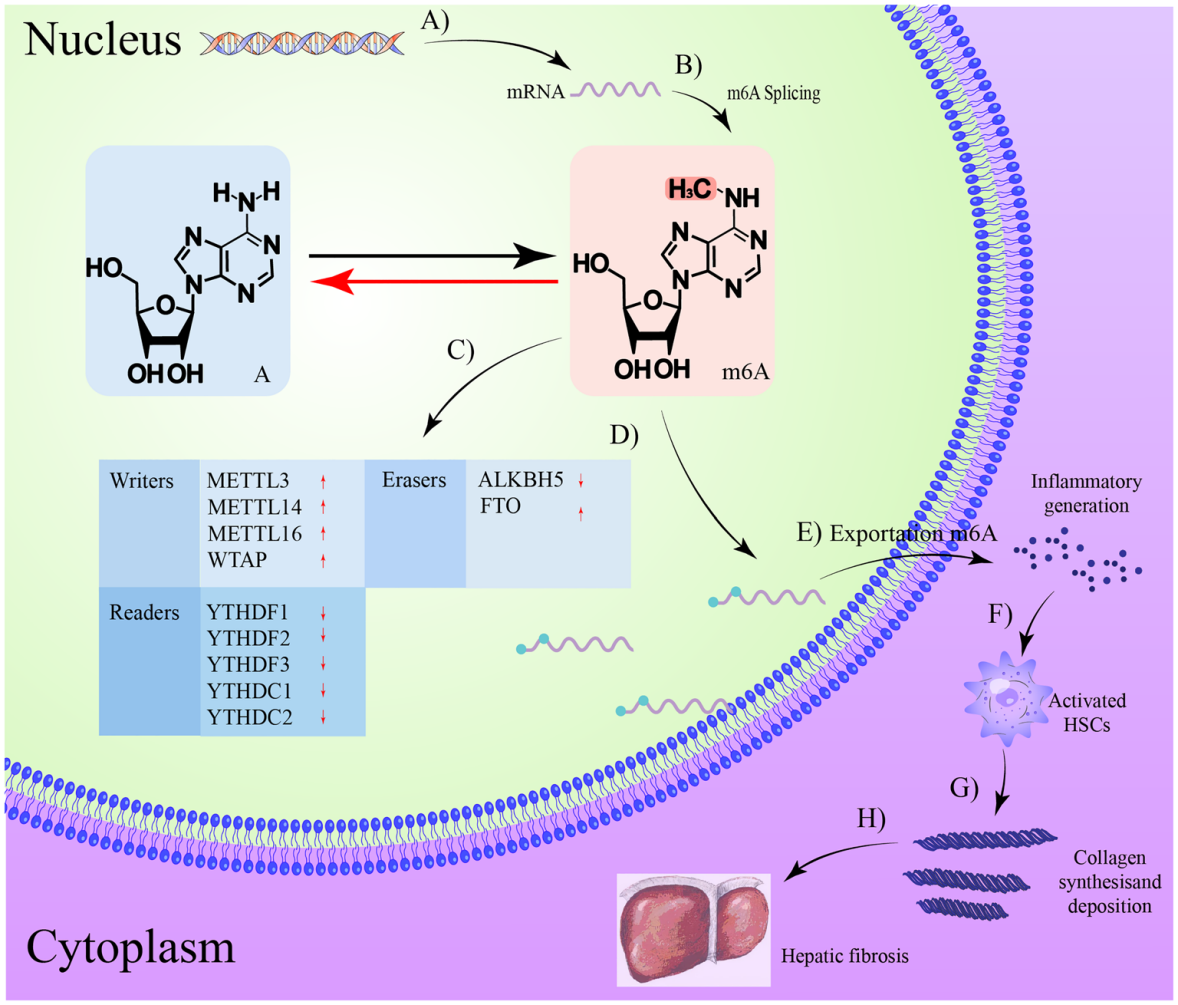
M6A methylation is involved in the progression of hepatic fibrosis. **A** Transcription of DNA into mRNA; **B** mRNA undergoes m6A modification; **C** changes in m6A modifying enzymes; **D** m6A modified mRNA; **E** stimulation of inflammatory mediator secretion; **F** stimulation of HSCs activation; **G** ECM production and accumulation; **H** fibrosis onset

RNA methylations are involved in the pathogenesis of HF, influencing disease onset and progression, and may be a new diagnostic biomarker and target for disease treatment (Table 1), (Fig. 3).

**Table 1:** m6A methylation related enzymes in HF

Diseases and cell types	Enzymes	Dysregulation	Functions	Targets	References
CCL4-induced mouse and M1-polarized macrophages	METTL3	Up-regulated	Stimulating pyroptosis and inflammation of macrophages, aggravates liver fibrosis	MALAT1/PTBP1/ USP8/TAK1	[100]
The activated HSC	METTL3	Up-regulated	Promotes the expression of fibrosis related genes	Lats2	[101]
PDGF-BB-induced activated HSC-T6	METTL3	Up-regulated	Promotes HSC activation	ASIC1a	[102]
High-fat diet induced rats	METTL3/14	Up-regulated	Promote the transition of NASH to HF	TGF-β1	[103]
Chronic corticosterone stimulation of chicken liver	METTL3	Up-regulated	Induces fibrosis in chicken	Heat shock proteins	[104]
A model of NAFLD in mice with type 2 diabetes mellitus	METTL3/14	Up-regulated	Promote NAFLD conversion to fibrosis	ACLY and SCD1	[105]
CCL4-induced rat and TGF-β1 treated HSC	WTAP	Up-regulated	Promotes HSC activation	Ptch1	[106]
Tissue of patients with mild and severe fibrosis	METTL16	Up-regulated	Regulating the m6A level and expression of HLA-DPB1	HLA-DPB1	[107]
Tissues of patients with liver cirrhosis complicated with HCC treated with sorafenib and CCL4-induced mouse	YTHDF1	Down-regulated	Triggers autophagy activation, and enhances HSC ferroptosis	BECN1	[108]
Mice were induced by a mixture of CCL4 and olive oil (1:9)	FTO	Up-regulated	Triggers autophagy activation, and enhances HSC ferroptosis	BECN1	[109]
TGF-β1 treated HSC	ALKBH5	Down-regulated	Improved liver fibrosis and inhibited HSC activation	PTCH1	[110]
Chronically HBV infected cases	ALKBH5	Up-regulated	ALKBH5 interacting with macrophage and WTAP interacting with nature killer T cells to promote hepatic fibrosis	Macrophage and killer T cells	[111]
	WTAP				
Liver tissue from patients with chronic fibrosis and CCL4-induced mouse	YTHDF3	Down-regulated	Promotes HSC activation	PRDX3	[112]

## M6A modification in hepatic fibrosis

### Methylation transferases in hepatic fibrosis

Fan et al. [98] performed a systematic assessment of genome-wide m6A modifications and mRNA expression in the liver by m6A-seq and RNA-seq. The results showed that 3315 genes had significantly altered m6A levels, of which 2498 were hypermethylated and 817 were hypomethylated. These differentially expressed m6A genes were closely associated with biological processes such as endoplasmic reticulum stress response, PPAR signaling pathway, and TGF-β signaling pathway. In addition, the methyltransferase WTAP, the demethylase ALKBH5, and the m6A binding protein YTHDF1, which are m6A regulatory enzymes, were all shown to be significantly down-regulated in HF.

M6A methylation is critical for regulating the progression and reversal of HF. In HF progression, differential m6A methylation was associated with oxidative stress and cytochrome metabolic processes. In contrast, during HF reversal, differential m6A methylation is associated with immune response and apoptosis-related processes [99].

In the HF model, up-regulated of METTL3 increased MALAT1 levels through m6A modification. MALAT1 directly interacted with PTBP1 to decrease USP8 levels. Down-regulated USP8 further promotes macrophage pyrosis and inflammation by affecting the ubiquitination and protein stability of TAK1. The METTL3/MALAT1/PTBP1/USP8/TAK1 axis can cause macrophage pyrosis and inflammation and promote the progression of HF. Therefore, targeting the various components of this axis may be beneficial in the treatment of HF [100].

METTL3 knockdown significantly alleviates HF by inhibiting HSCs activation through control of the Hippo/YAP signaling pathway. Mechanistically, METTL3 deletion reduces the deposition of m6A on Lats2 mRNA transcripts and slows their degradation. Up-regulation of Lats2 promotes phosphorylation of the downstream transcription factor YAP, inhibits YAP nuclear translocation, and reduces the expression of pro-fibrotic genes [101]. Acid-sensitive ion channel 1a (ASIC1a) is significantly up-regulated in HF. ASIC1a regulates miR-350 expression through METTL3-mediated m6A modifications. The regulated miR-350 targets SPRY2 and further promotes HF through the PI3K/KT and ERK pathways. When ASIC1a is knocked down, HSCs activation and HF are inhibited, and m6A modification levels and miR-350 expression are reduced [102].

During NASH to HF, post-transcriptional regulation of TGF-β1 is determined by m6A modification. The NF-κB pathway promotes m6A methylation of TGF-β1 mRNA through activation of METTL3/METTL14, which exacerbates TGF-β1-mediated HSCs activation and promotes the transition from NASH to HF [103]. In addition, chronic corticosteroid (CORT)-induced fibrosis in chickens may be associated with up-regulated METTL3, promoting heat shock protein (HSP) m6A methylation, and inhibiting the protective effects of HSP [104].

Overexpression of METTL14 in NAFLD models and hepatocellular carcinoma samples mediates m6A methylation of ACLY and SCD1, leading to upregulation of ACLY and SCD1 proteins, triglyceride and cholesterol production, and lipid droplet accumulation [105]. N-acetyl-seryl-aspartyl-lysyl-proline (AcSDKP) is an endogenous tetrapeptide with antifibrotic effects. AcSDKP downregulates the expression of the methyltransferase WTAP, leading to a significant decrease in the stability of Ptch1 mRNA in the Hedgehog pathway, which exerts antifibrotic effects [106]. Chronic hepatitis B (CHB)-related genes such as HLA-DPA1 and HLA-DPB1 are significantly differentially expressed in the tissues of patients with mild and severe HF. Furthermore, silencing METTL16 suppressed the expression of m6A and HLA-DPB1. This suggests that METTL16 may be a new target for the diagnosis and treatment of CHB fibrosis [107].

### Demethylation enzymes in hepatic fibrosis

Ferroptosis is considered a novel and effective approach to clearing HSCs to alleviate HF. Clinical treatment with sorafenib and erastin promotes HSCs ferroptosis by inhibiting BECN1 mRNA stabilization through YTHDF1. Dihydroartemisinin inhibits FTO activation. Down-regulated FTO inhibits HSCs activation by inducing HSCs ferroptosis. This reveals a new molecular mechanism of ferroptosis and may identify m6A modification as a potential target for HF therapy [108, 109].

The expression levels of ALKBH5 and PTCH1 are significantly down-regulated in HF. ALKBH5 upregulates PTCH1 expression by mediating m6A demethylation, leading to the inactivation of the hedgehog pathway. This reduced α-SMA and type I collagen levels improved HF and inhibited HSCs activation [110]. ALKBH5 and WETP were significantly up-regulated in patients with HF. Immune cell infiltration and fibrosis occur in HBV-infected livers, and ALKBH5 interaction with macrophages and WETP interaction with natural killer T cells are thought to be key points of m6A modification regulation in HF progression [111].

### Methylation binding proteins in hepatic fibrosis

YTHDF3 is significantly up-regulated in the liver tissue of chronic fibrosis patients and CCl4-induced mice. Peroxiredoxin 3 (PRDX3) acts as a major regulator of mitochondrial oxidative stress and is hepatoprotective. Knockdown of PRDX3 exacerbates HF and HSCs activation, while HSC-specific PRDX3 overexpression attenuates HF. Translation of PRDX3 mRNA is regulated by YTHDF3-mediated m6A modification, and PRDX3 can inhibit HSCs activation by regulating the mitochondrial ROS/TGF-β1/Smad2/3 pathway [112].

The methyltransferases METTLl3, METTL14, and the demethylase FTO are significantly up-regulated in patients with NAFLD. However, the expression of the m6A binding proteins YTHDC1, YTHDC2, and IGF2BP1 was reduced. MYC abnormalities are thought to be a key link in the regulation of NAFLD by m6A. Higher MYC mRNA levels were accompanied by higher levels of HDL cholesterol and unsaturated fatty acid ratios, as well as lower adiposity, glucose and transaminases. This suggests that aberrant regulation of m6A methylation leads to steatosis and fibrosis and influences the development of NAFLD, of which MYC may be a potential target [113].

### Other methylation modifications and hepatic fibrosis

M6Am is located precisely at the nucleotide of the first eukaryotic transcription adjacent to the cap structure of the mRNA, and its function is mainly to improve the stability of the mRNA. M5C has rich and highly dynamic properties, which play a role in regulating intracellular RNA metabolism and related functions. M7G modification is a self-positively charged RNA methylation modification in which methyl groups are catalyzed by methyltransferase to bind to the 7th nitrogen atom of RNA guanine. M1A methylation mainly occurs in rRNA and tRNA and is involved in the maintenance of RNA tertiary structure and affects the efficiency of protein translation. M6Am, m5C, m7G, and m1A may be closely related to the occurrence of HF, but their relationship has not been reported so far.

## Conclusion and prospect

This work reviews the mechanisms, structural molecules, and functional biology of each of the five types of RNA methylation, special the role of RNA methylation in HF. In RNA methylations, methyltransferases are responsible for catalyzing RNA to undergo methylation modifications; demethylases delete these modifications; and methylation-binding proteins affect mRNA splicing, export, translation, and degradation. In addition, m6A affects the splicing and processing of miRNA precursors. The absence of METTL3 reduced the binding of the binding protein DGCR8 to pri-miRNA and resulted in the accumulation of pri-miRNA and the decrease of mature miRNA. Currently, the m6A disorder in HF and its impact on pathogenesis are being slowly revealed. However, the roles of m5C, m1A, m6Am, and m7G in HF have not been reported.

Combined with the current research status of RNA methylation in HF, the mechanism of RNA methylation in HF may be the abnormal expression of various enzymes involved in the process of RNA methylation. These aberrantly expressed enzymes interact with downstream transcription factors to influence the mRNA synthesis process and promote or inhibit the development of HSCs. Although RNA methylation has been the focus of many studies in recent years, our understanding of it is far from complete. The specificity between the location and level of methylation on the RNA sequence and the reading protein remains unknown. The mutual competitive cooperation between different RNA methylation-modifying enzymes needs further elucidation.

Traditional Chinese medicine compounds are characterized by multiple components, multiple targets, and multiple pathways of action. Therefore, it has its own characteristics and advantages in the prevention and treatment of complex diseases [114]. This advantage provides the possibility for herbal compounds to interact with RNA methylation-modifying enzymes. Whether key compounds in plants affect disease progression by binding or inhibiting methylation modifying-enzymes deserves further investigation.

Wilson's disease (WD) is an autosomal recessive genetic disorder. Mutations in the causative gene ATP7B lead to copper deposition in the liver, brain, and cornea, resulting in impaired Cu^2+^ metabolism and damage to the corresponding tissues and organs [115]. The clinical symptoms of WD are mainly distinguished by liver and nervous system manifestations, and almost all WD patients with liver damage have HF [116, 117]. Our group found that the active ingredient of the Chinese herbal compound Gandouling has better binding ability with m6A methylation transferase through molecular docking. The next step in our study of HF in WD will be m6A modification, which may provide new prospects for WD therapy.

## Data Availability

Not applicable.

## Abbreviations

AcSDKP: N-acetyl-seryl-aspartyl-lysyl-proline
ALKBH5: Human AlkB homolog 5
AngII: Angiotensin II
ASIC1a: Acid-sensitive ion channel 1a
CCL4: Carbon tetrachloride
CHB: Chronic hepatitis B
ECM: Extracellular matrix
FTO: Fat mass and obesity-associated protein
HF: Hepatic fibrosis
HSCs: Hepatic stellate cells
HSP: Heat shock protein
KCs: Kupffer cells
METTL3: Methyltransferase like 3
METTL14: Methyltransferase like 14
MFB: Myofibroblast
MMP: Matrix metalloproteinase
m1A: N1-methyladenosine
m5C: 5-Methylcytosine
m6A: N6-methyladenosine
m6Am: N6,2-O-dimethyladenosine
m7G: 7-Methylguanosine
NAFLD: Nonalcoholic fatty liver disease
NASH: Nonalcoholic steatohepatitis
Nrf2: Nuclear factor erythroid2-related factor 2
NSCLC: Non-small cell lung cancer
PDGF: Platelet-derived growth factor
PRDX3: Peroxiredoxin 3
ROS: Reactive oxygen species
TGF-β: Transforming growth factor beta
TIMP: Tissue inhibitor of metalloprotein-ase
WTAP: Wilms tumor 1 associated protein
YBX1: Y-box binding protein 1

## References

[1] Bataller R, Brenner DA (2005). Liver fibrosis. J Clin Invest.

[2] Tsuchida T, Friedman SL (2017). Mechanisms of hepatic stellate cell activation. Nat Rev Gastroenterol Hepatol.

[3] Zhang D, Zhang Y, Sun B (2022). The molecular mechanisms of liver fibrosis and its potential therapy in application. Int J Mol Sci.

[4] Parola M, Pinzani M (2019). Liver fibrosis: Pathophysiology, pathogenetic targets and clinical issues. Mol Aspects Med.

[5] Huang E, Peng N, Xiao F, Hu D, Wang X, Lu L (2020). The roles of immune cells in the pathogenesis of fibrosis. Int J Mol Sci.

[6] Koyama Y, Brenner DA (2017). Liver inflammation and fibrosis. J Clin Invest.

[7] Franco KGS, de Amorim FJR, Santos MA, Rollemberg CVV, de Oliveira FA, França AVC, Santos CNO, Magalhães LS, Cazzaniga RA, de Lima FS, Benevides L, Carregaro V, Silva JS, Brito HLF, Fernandes DA, da Silva M, Â, RP de Almeida, M Bezerra-Santos, AR de Jesus.  (2021). Association of IL-9, IL-10, and IL-17 cytokines with hepatic fibrosis in human schistosoma mansoni infection. Front Immunol.

[8] Preziosi ME, Singh S, Valore EV, Jung G, Popovic B, Poddar M, Nagarajan S, Ganz T, Monga SP (2017). Mice lacking liver-specific β-catenin develop steatohepatitis and fibrosis after iron overload. J Hepatol.

[9] Bertola A, Mathews S, Ki SH, Wang H, Gao B (2013). Mouse model of chronic and binge ethanol feeding (the NIAAA model). Nat Protoc.

[10] Luedde T, Kaplowitz N, Schwabe RF (2014). Cell death and cell death responses in liver disease: mechanisms and clinical relevance. Gastroenterology.

[11] Arunsan P, Ittiprasert W, Smout MJ, Cochran CJ, Mann VH, Chaiyadet S, Karinshak SE, Sripa B, Young ND, Sotillo J, Loukas A, Brindley PJ, Laha T (2019). Programmed knockout mutation of liver fluke granulin attenuates virulence of infection-induced hepatobiliary morbidity. Elife.

[12] Li X, Jin Q, Xu H, Zhang Z, Zhou H, Yan D, Li D, Gao P, Niu J (2017). Chronic hepatitis B patients with high liver fibrosis levels should receive antiviral treatment. Exp Ther Med.

[13] Jensen T, Abdelmalek MF, Sullivan S, Nadeau KJ, Green M, Roncal C, Nakagawa T, Kuwabara M, Sato Y, Kang DH, Tolan DR, Sanchez-Lozada LG, Rosen HR, Lanaspa MA, Diehl AM, Johnson RJ (2018). Fructose and sugar: a major mediator of non-alcoholic fatty liver disease. J Hepatol.

[14] Saltiel AR, Olefsky JM (2017). Inflammatory mechanisms linking obesity and metabolic disease. J Clin Invest.

[15] Ratziu V, Giral P, Charlotte F, Bruckert E, Thibault V, Theodorou I, Khalil L, Turpin G, Opolon P, Poynard T (2000). Liver fibrosis in overweight patients. Gastroenterology.

[16] Shay JES, Hamilton JP (2018). Hepatic fibrosis: avenues of investigation and clinical implications. Clin Liver Dis.

[17] Furlan M, de Pretis S, Pelizzola M (2021). Dynamics of transcriptional and post-transcriptional regulation. Brief Bioinform.

[18] Zanzoni A, Spinelli L, Ribeiro DM, Tartaglia GG, Brun C (2019). Post-transcriptional regulatory patterns revealed by protein-RNA interactions. Sci Rep.

[19] Moore MJ (2005). From birth to death: the complex lives of eukaryotic mRNAs. Science.

[20] Zhou W, Wang X, Chang J, Cheng C, Miao C (2022). The molecular structure and biological functions of RNA methylation, with special emphasis on the roles of RNA methylation in autoimmune diseases. Crit Rev Clin Lab Sci.

[21] Xiao W, Adhikari S, Dahal U, Chen YS, Hao YJ, Sun BF, Sun HY, Li A, Ping XL, Lai WY, Wang X, Ma HL, Huang CM, Yang Y, Huang N, Jiang GB, Wang HL, Zhou Q, Wang XJ, Zhao YL, Yang YG (2016). Nuclear m(6)A reader YTHDC1 regulates mRNA splicing. Mol Cell.

[22] Roundtree IA, Luo GZ, Zhang Z, Wang X, Zhou T, Cui Y, Sha J, Huang X, Guerrero L, Xie P, He E, Shen B, He C (2017). YTHDC1 mediates nuclear export of N6-methyladenosine methylated mRNAs. Elife.

[23] Wang X, Lu Z, Gomez A, Hon GC, Yue Y, Han D, Fu Y, Parisien M, Dai Q, Jia G, Ren B, Pan T, He C (2014). N6-methyladenosine-dependent regulation of messenger RNA stability. Nature.

[24] Meyer KD, Patil DP, Zhou J, Zinoviev A, Skabkin MA, Elemento O, Pestova TV, Qian SB, Jaffrey SR (2015). 5' UTR m(6)A promotes cap-independent translation. Cell.

[25] Wang X, Zhao BS, Roundtree IA, Lu Z, Han D, Ma H, Weng X, Chen K, Shi H, He C (2015). N(6)-methyladenosine modulates messenger RNA translation efficiency. Cell.

[26] Jiang X, Liu B, Nie Z, Duan L, Xiong Q, Jin Z, Yang C, Chen Y (2021). The role of m6A modification in the biological functions and diseases. Sign Transduct Target Ther.

[27] Song H, Zhang J, Liu B, Xu J, Cai B, Yang H, Straube J, Yu X, Ma T (2022). Biological roles of RNA m(5)C modification and its implications in cancer immunotherapy. Biomark Res.

[28] Naim A, Pan Q, Baig MS (2017). Matrix metalloproteinases (MMPs) in liver diseases. J Clin Exp Hepatol.

[29] Duarte S, Baber J, Fujii T, Coito AJ (2015). Matrix metalloproteinases in liver injury, repair and fibrosis. Matrix Biol.

[30] Liu N, Feng J, Lu X, Yao Z, Liu Q, Lv Y, Han Y, Deng J, Zhou Y (2019). Isorhamnetin inhibits liver fibrosis by reducing autophagy and inhibiting extracellular matrix formation via the TGF-β1/Smad3 and TGF-β1/p38 MAPK pathways. Med Inflamm.

[31] Ciceu A, Balta C, Herman H, Gharbia S, Ignat SR, Dinescu S, Váradi J, Fenyvesi F, Gyöngyösi S, Hermenean A, Costache M (2021). Complexation with random methyl-β-cyclodextrin and (2-hidroxypropyl)-β-cyclodextrin enhances in vivo anti-fibrotic and anti-inflammatory effects of chrysin via the inhibition of NF-κB and TGF-β1/smad signaling pathways and modulation of hepatic pro/anti-fibrotic miRNA. Int J Mol Sci.

[32] Kong D, Zhang Z, Chen L, Huang W, Zhang F, Wang L, Wang Y, Cao P, Zheng S (2020). Curcumin blunts epithelial-mesenchymal transition of hepatocytes to alleviate hepatic fibrosis through regulating oxidative stress and autophagy. Redox Biol.

[33] Gupta G, Khadem F, Uzonna JE (2019). Role of hepatic stellate cell (HSC)-derived cytokines in hepatic inflammation and immunity. Cytokine.

[34] Saeed A, Bartuzi P, Heegsma J, Dekker D, Kloosterhuis N, de Bruin A, Jonker JW, van de Sluis B, Faber KN (2021). Impaired hepatic vitamin A metabolism in NAFLD mice leading to vitamin A accumulation in hepatocytes. Cell Mol Gastroenterol Hepatol.

[35] Lee UE, Friedman SL (2011). Mechanisms of hepatic fibrogenesis. Best Pract Res Clin Gastroenterol.

[36] Khomich O, Ivanov AV, Bartosch B (2019). Metabolic hallmarks of hepatic stellate cells in liver fibrosis. Cells.

[37] Hinz B, Lagares D (2020). Evasion of apoptosis by myofibroblasts: a hallmark of fibrotic diseases. Nat Rev Rheumatol.

[38] Krenkel O, Hundertmark J, Ritz TP, Weiskirchen R, Tacke F (2019). Single cell RNA sequencing identifies subsets of hepatic stellate cells and myofibroblasts in liver fibrosis. Cells.

[39] Morikawa M, Derynck R, Miyazono K (2016). TGF-β and the TGF-β family: context-dependent roles in cell and tissue physiology. Cold Spring Harb Perspect Biol.

[40] Kocabayoglu P, Lade A, Lee YA, Dragomir AC, Sun X, Fiel MI, Thung S, Aloman C, Soriano P, Hoshida Y, Friedman SL (2015). β-PDGF receptor expressed by hepatic stellate cells regulates fibrosis in murine liver injury, but not carcinogenesis. J Hepatol.

[41] Wang X, Gao Y, Li Y, Huang Y, Zhu Y, Lv W, Wang R, Gou L, Cheng C, Feng Z, Xie J, Tian J, Yao R (2020). Roseotoxin B alleviates cholestatic liver fibrosis through inhibiting PDGF-B/PDGFR-β pathway in hepatic stellate cells. Cell Death Dis.

[42] Wang Y, Wang P, Yu Y, Huang E, Yao Y, Guo D, Peng H, Tian B, Zheng Q, Jia M, Wang J, Wu X, Cheng J, Liu H, Wang QK, Xu C (2023). Hepatocyte Ninjurin2 promotes hepatic stellate cell activation and liver fibrosis through the IGF1R/EGR1/PDGF-BB signaling pathway. Metab Clin Exp.

[43] Borkham-Kamphorst E, Weiskirchen R (2016). The PDGF system and its antagonists in liver fibrosis. Cytokine Growth Factor Rev.

[44] Li P, He K, Li J, Liu Z, Gong J (2017). The role of Kupffer cells in hepatic diseases. Mol Immunol.

[45] Liu C, Tao Q, Sun M, Wu JZ, Yang W, Jian P, Peng J, Hu Y, Liu C, Liu P (2010). Kupffer cells are associated with apoptosis, inflammation and fibrotic effects in hepatic fibrosis in rats. Lab Invest.

[46] Tacke F, Zimmermann HW (2014). Macrophage heterogeneity in liver injury and fibrosis. J Hepatol.

[47] Ye L, He S, Mao X, Zhang Y, Cai Y, Li S (2020). Effect of hepatic macrophage polarization and apoptosis on liver ischemia and reperfusion injury during liver transplantation. Front Immunol.

[48] Fabregat I, Caballero-Díaz D (2018). Transforming growth factor-β-induced cell plasticity in liver fibrosis and hepatocarcinogenesis. Front Oncol.

[49] Wang Y, Zhang C (2019). The roles of liver-resident lymphocytes in liver diseases. Front Immunol.

[50] Xu J, Liu X, Koyama Y, Wang P, Lan T, Kim IG, Kim IH, Ma HY, Kisseleva T (2014). The types of hepatic myofibroblasts contributing to liver fibrosis of different etiologies. Front Pharmacol.

[51] Kukla M, Mazur W, Bułdak RJ, Zwirska-Korczala K (2011). Potential role of leptin, adiponectin and three novel adipokines–visfatin, chemerin and vaspin–in chronic hepatitis. Mol Med.

[52] Saxena NK, Anania FA (2015). Adipocytokines and hepatic fibrosis. Trends Endocrinol Metab.

[53] AlQudah M, Hale TM, Czubryt MP (2020). Targeting the renin-angiotensin-aldosterone system in fibrosis. Matrix Biol.

[54] Ning ZW, Luo XY, Wang GZ, Li Y, Pan MX, Yang RQ, Ling XG, Huang S, Ma XX, Jin SY, Wang D, Li X (2017). MicroRNA-21 mediates angiotensin II-induced liver fibrosis by activating NLRP3 inflammasome/IL-1β axis via targeting smad7 and spry1. Antioxid Redox Signal.

[55] An SY, Petrescu AD, DeMorrow S (2021). Targeting certain interleukins as novel treatment options for liver fibrosis. Front Pharmacol.

[56] Kagan P, Sultan M, Tachlytski I, Safran M, Ben-Ari Z (2017). Both MAPK and STAT3 signal transduction pathways are necessary for IL-6-dependent hepatic stellate cells activation. PLoS ONE.

[57] Raabe J, Kaiser KM, ToVinh M, Finnemann C, Lutz P, Hoffmeister C, Bischoff J, Goeser F, Kaczmarek DJ, Glowka TR, Manekeller S, Charpentier A, Langhans B, Nischalke HD, Toma M, Strassburg CP, Spengler U, Abdallah AT, Krämer B, Nattermann J (2023). Identification and characterisation of a hepatic IL-13 producing ILC3-like population potentially involved in liver fibrosis. Hepatology.

[58] Kartasheva-Ebertz D, Gaston J, Lair-Mehiri L, Mottez E, Buivan TP, Massault PP, Scatton O, Gaujoux S, Vaillant JC, Pol S, Lagaye S (2022). IL-17A in Human liver: significant source of inflammation and trigger of liver fibrosis initiation. Int J Mol Sci.

[59] Wree A, McGeough MD, Inzaugarat ME, Eguchi A, Schuster S, Johnson CD, Peña CA, Geisler LJ, Papouchado BG, Hoffman HM, Feldstein AE (2018). NLRP3 inflammasome driven liver injury and fibrosis: roles of IL-17 and TNF in mice. Hepatology.

[60] He D, Xu H, Zhang H, Tang R, Lan Y, Xing R, Li S, Christian E, Hou Y, Lorello P, Caldarone B, Ding J, Nguyen L, Dionne D, Thakore P, Schnell A, Huh JR, Rozenblatt-Rosen O, Regev A, Kuchroo VK (2022). Disruption of the IL-33-ST2-AKT signaling axis impairs neurodevelopment by inhibiting microglial metabolic adaptation and phagocytic function. Immunity.

[61] Xiang X, Feng D, Hwang S, Ren T, Wang X, Trojnar E, Matyas C, Mo R, Shang D, He Y, Seo W, Shah VH, Pacher P, Xie Q, Gao B (2020). Interleukin-22 ameliorates acute-on-chronic liver failure by reprogramming impaired regeneration pathways in mice. J Hepatol.

[62] Wu Y, Min J, Ge C, Shu J, Tian D, Yuan Y, Zhou D (2020). Interleukin 22 in liver injury, inflammation and cancer. Int J Biol Sci.

[63] Su SB, Qin SY, Xian XL, Huang FF, Huang QL, ZhangDi HJ, Jiang HX (2021). Interleukin-22 regulating Kupffer cell polarization through STAT3/Erk/Akt crosstalk pathways to extenuate liver fibrosis. Life Sci.

[64] Rodríguez MJ, Sabaj M, Tolosa G, Herrera Vielma F, Zúñiga MJ, González DR, Zúñiga-Hernández J (2021). Maresin-1 prevents liver fibrosis by targeting Nrf2 and NF-κB reducing oxidative stress and inflammation. Cells.

[65] Gallego P, Luque-Sierra A, Falcon G, Carbonero P, Grande L, Bautista JD, Martín F, Del Campo JA (2021). White button mushroom extracts modulate hepatic fibrosis progression, inflammation, and oxidative stress in vitro and in LDLR-/- mice. Foods.

[66] Zhou J, Zheng Q, Chen Z (2022). The Nrf2 pathway in liver diseases. Front Cell Dev Biol.

[67] Shin MR, Lee JA, Kim M, Lee S, Oh M, Moon J, Nam JW, Choi H, Mun YJ, Roh SS (2021). Gardeniae fructus attenuates thioacetamide-induced liver fibrosis in mice via both AMPK/SIRT1/NF-κB pathway and Nrf2 signaling. Antioxidants.

[68] Khadrawy SM, Mohamed HM, Mahmoud AM (2021). Mesenchymal stem cells ameliorate oxidative stress, inflammation, and hepatic fibrosis via Nrf2/HO-1 signaling pathway in rats. Environ Sci Pollut Res Int.

[69] Desrosiers R, Friderici K, Rottman F (1974). Identification of methylated nucleosides in messenger RNA from Novikoff hepatoma cells. Proc Natl Acad Sci U S A.

[70] Oerum S, Meynier V, Catala M, Tisné C (2021). A comprehensive review of m6A/m6Am RNA methyltransferase structures. Nucleic Acids Res.

[71] Zhang T, Zhang SW, Zhang SY, Gao SJ, Chen Y, Huang Y (2021). m6A-express: uncovering complex and condition-specific m6A regulation of gene expression. Nucleic Acids Res.

[72] Liu J, Yue Y, Han D, Wang X, Fu Y, Zhang L, Jia G, Yu M, Lu Z, Deng X, Dai Q, Chen W, He C (2014). A METTL3-METTL14 complex mediates mammalian nuclear RNA N6-adenosine methylation. Nat Chem Biol.

[73] Wang X, Feng J, Xue Y, Guan Z, Zhang D, Liu Z, Gong Z, Wang Q, Huang J, Tang C, Zou T, Yin P (2016). Structural basis of N(6)-adenosine methylation by the METTL3-METTL14 complex. Nature.

[74] Akichika S, Hirano S, Shichino Y, Suzuki T, Nishimasu H, Ishitani R, Sugita A, Hirose Y, Iwasaki S, Nureki O, Suzuki T (2019). Cap-specific terminal N (6)-methylation of RNA by an RNA polymerase II-associated methyltransferase. Science.

[75] Keith JM, Ensinger MJ, Moss B (1978). HeLa cell RNA (2'-O-methyladenosine-N6-)-methyltransferase specific for the capped 5'-end of messenger RNA. J Biol Chem.

[76] Sun H, Li K, Zhang X, Liu J, Zhang M, Meng H, Yi C (2021). m(6)Am-seq reveals the dynamic m(6)Am methylation in the human transcriptome. Nat Commun.

[77] Bohnsack KE, Höbartner C, Bohnsack MT (2019). Eukaryotic 5-methylcytosine (m^5^C) RNA methyltransferases: mechanisms, cellular functions, and links to disease. Genes.

[78] Zhao BS, Roundtree IA, He C (2017). Post-transcriptional gene regulation by mRNA modifications. Nat Rev Mol Cell Biol.

[79] Zhou J, Wan J, Gao X, Zhang X, Jaffrey SR, Qian SB (2015). Dynamic m(6)A mRNA methylation directs translational control of heat shock response. Nature.

[80] Malbec L, Zhang T, Chen YS, Zhang Y, Sun BF, Shi BY, Zhao YL, Yang Y, Yang YG (2019). Dynamic methylome of internal mRNA N(7)-methylguanosine and its regulatory role in translation. Cell Res.

[81] Lin S, Liu Q, Lelyveld VS, Choe J, Szostak JW, Gregory RI (2018). Mettl1/Wdr4-Mediated m(7)G tRNA methylome Is required for normal mRNA translation and embryonic stem cell self-renewal and differentiation. Mol Cell.

[82] RajBhandary UL, Stuart A, Faulkner RD, Chang SH, Khorana HG (1966). Nucleotide sequence studies on yeast phenylalanine sRNA. Cold Spring Harb Symp Quant Biol.

[83] Fukuda H, Chujo T, Wei FY, Shi SL, Hirayama M, Kaitsuka T, Yamamoto T, Oshiumi H, Tomizawa K (2021). Cooperative methylation of human tRNA3Lys at positions A58 and U54 drives the early and late steps of HIV-1 replication. Nucleic Acids Res.

[84] Liu F, Clark W, Luo G, Wang X, Fu Y, Wei J, Wang X, Hao Z, Dai Q, Zheng G, Ma H, Han D, Evans M, Klungland A, Pan T, He C (2016). ALKBH1-mediated tRNA demethylation regulates translation. Cell.

[85] Fendt SM, Frezza C, Erez A (2020). Targeting metabolic plasticity and flexibility dynamics for cancer therapy. Cancer Discov.

[86] Delaunay S, Pascual G, Feng B, Klann K, Behm M, Hotz-Wagenblatt A, Richter K, Zaoui K, Herpel E, Münch C, Dietmann S, Hess J, Benitah SA, Frye M (2022). Mitochondrial RNA modifications shape metabolic plasticity in metastasis. Nature.

[87] Nakano S, Suzuki T, Kawarada L, Iwata H, Asano K, Suzuki T (2016). NSUN3 methylase initiates 5-formylcytidine biogenesis in human mitochondrial tRNA(Met). Nat Chem Biol.

[88] Su R, Dong L, Li Y, Gao M, He PC, Liu W, Wei J, Zhao Z, Gao L, Han L, Deng X, Li C, Prince E, Tan B, Qing Y, Qin X, Shen C, Xue M, Zhou K, Chen Z, Xue J, Li W, Qin H, Wu X, Sun M, Nam Y, Chen CW, Huang W, Horne D, Rosen ST, He C, Chen J (2022). METTL16 exerts an m(6)A-independent function to facilitate translation and tumorigenesis. Nat Cell Biol.

[89] Chokkalla AK, Mehta SL, Vemuganti R (2020). Epitranscriptomic regulation by m(6)A RNA methylation in brain development and diseases. J Cereb Blood Flow Metab.

[90] Yoon KJ, Ringeling FR, Vissers C, Jacob F, Pokrass M, Jimenez-Cyrus D, Su Y, Kim NS, Zhu Y, Zheng L, Kim S, Wang X, Doré LC, Jin P, Regot S, Zhuang X, Canzar S, He C, Ming GL, Song H (2017). Temporal control of mammalian cortical neurogenesis by m(6)A methylation. Cell.

[91] Shi H, Zhang X, Weng YL, Lu Z, Liu Y, Lu Z, Li J, Hao P, Zhang Y, Zhang F, Wu Y, Delgado JY, Su Y, Patel MJ, Cao X, Shen B, Huang X, Ming GL, Zhuang X, Song H, He C, Zhou T (2018). m(6)A facilitates hippocampus-dependent learning and memory through YTHDF1. Nature.

[92] De Jesus DF, Zhang Z, Kahraman S, Brown NK, Chen M, Hu J, Gupta MK, He C, Kulkarni RN (2019). m(6)A mRNA methylation regulates human β-cell biology in physiological states and in type 2 diabetes. Nat Metab.

[93] Wu Y, Xie L, Wang M, Xiong Q, Guo Y, Liang Y, Li J, Sheng R, Deng P, Wang Y, Zheng R, Jiang Y, Ye L, Chen Q, Zhou X, Lin S, Yuan Q (2018). Mettl3-mediated m(6)A RNA methylation regulates the fate of bone marrow mesenchymal stem cells and osteoporosis. Nat Commun.

[94] Zhong X, Yu J, Frazier K, Weng X, Li Y, Cham CM, Dolan K, Zhu X, Hubert N, Tao Y, Lin F, Martinez-Guryn K, Huang Y, Wang T, Liu J, He C, Chang EB, Leone V (2018). Circadian clock regulation of hepatic lipid metabolism by modulation of m(6)A mRNA methylation. Cell Rep.

[95] Alarcón CR, Lee H, Goodarzi H, Halberg N, Tavazoie SF (2015). N6-methyladenosine marks primary microRNAs for processing. Nature.

[96] Weng H, Huang H, Wu H, Qin X, Zhao BS, Dong L, Shi H, Skibbe J, Shen C, Hu C, Sheng Y, Wang Y, Wunderlich M, Zhang B, Dore LC, Su R, Deng X, Ferchen K, Li C, Sun M, Lu Z, Jiang X, Marcucci G, Mulloy JC, Yang J, Qian Z, Wei M, He C, Chen J (2018). METTL14 Inhibits hematopoietic stem/progenitor differentiation and promotes leukemogenesis via mRNA m(6)A modification. Cell Stem Cell.

[97] Shi Y, Fan S, Wu M, Zuo Z, Li X, Jiang L, Shen Q, Xu P, Zeng L, Zhou Y, Huang Y, Yang Z, Zhou J, Gao J, Zhou H, Xu S, Ji H, Shi P, Wu DD, Yang C, Chen Y (2019). YTHDF1 links hypoxia adaptation and non-small cell lung cancer progression. Nat Commun.

[98] Fan C, Ma Y, Chen S, Zhou Q, Jiang H, Zhang J, Wu F (2021). Comprehensive analysis of the transcriptome-wide m6A methylation modification difference in liver fibrosis mice by high-throughput m6A sequencing. Front Cell Dev Biol.

[99] Cui Z, Huang N, Liu L, Li X, Li G, Chen Y, Wu Q, Zhang J, Long S, Wang M, Sun F, Shi Y, Pan Q (2020). Dynamic analysis of m6A methylation spectroscopy during progression and reversal of hepatic fibrosis. Epigenomics.

[100] Shu B, Zhou YX, Li H, Zhang RZ, He C, Yang X (2021). The METTL3/MALAT1/PTBP1/USP8/TAK1 axis promotes pyroptosis and M1 polarization of macrophages and contributes to liver fibrosis. Cell Death Discov.

[101] Li Y, Kang X, Zhou Z, Pan L, Chen H, Liang X, Chu J, Dong S, Liu C, Yu S, Tu D, Zhang Y, Ge M, Chen W, Xu Y, Zhang Q (2022). The m(6)A methyltransferase Mettl3 deficiency attenuates hepatic stellate cell activation and liver fibrosis. Mol Ther.

[102] Zhu Y, Pan X, Du N, Li K, Hu Y, Wang L, Zhang J, Liu Y, Zuo L, Meng X, Hu C, Wu X, Jin J, Wu W, Chen X, Wu F, Huang Y (2020). ASIC1a regulates miR-350/SPRY2 by N(6) -methyladenosine to promote liver fibrosis. Faseb j.

[103] Feng Y, Dong H, Sun B, Hu Y, Yang Y, Jia Y, Jia L, Zhong X, Zhao R (2021). METTL3/METTL14 transactivation and m(6)A-dependent TGF-β1 translation in activated kupffer cells. Cell Mol Gastroenterol Hepatol.

[104] Feng Y, Hu Y, Hou Z, Sun Q, Jia Y, Zhao R (2020). Chronic corticosterone exposure induces liver inflammation and fibrosis in association with m(6)A-linked post-transcriptional suppression of heat shock proteins in chicken. Cell Stress Chaperones.

[105] Yang Y, Cai J, Yang X, Wang K, Sun K, Yang Z, Zhang L, Yang L, Gu C, Huang X, Wang Z, Zhu X (2022). Dysregulated m6A modification promotes lipogenesis and development of non-alcoholic fatty liver disease and hepatocellular carcinoma. Mol Ther.

[106] Wei A, Zhao F, Hao A, Liu B, Liu Z (2022). N-acetyl-seryl-aspartyl-lysyl-proline (AcSDKP) mitigates the liver fibrosis via WTAP/m(6)A/Ptch1 axis through Hedgehog pathway. Gene.

[107] Gao H, Wang X, Ma H, Lin S, Zhang D, Wu W, Liao Z, Chen M, Li Q, Lin M, Li D (2022). METTL16 regulates m(6)A methylation on chronic hepatitis B associated gene HLA-DPB1 involved in liver fibrosis. Front Genet.

[108] Shen M, Li Y, Wang Y, Shao J, Zhang F, Yin G, Chen A, Zhang Z, Zheng S (2021). N(6)-methyladenosine modification regulates ferroptosis through autophagy signaling pathway in hepatic stellate cells. Redox Biol.

[109] Shen M, Guo M, Li Y, Wang Y, Qiu Y, Shao J, Zhang F, Xu X, Yin G, Wang S, Chen A, Zhang Z, Zheng S (2022). m(6)A methylation is required for dihydroartemisinin to alleviate liver fibrosis by inducing ferroptosis in hepatic stellate cells. Free Radic Biol Med.

[110] Yang JJ, Wang J, Yang Y, Yang Y, Li J, Lu D, Lu C (2022). ALKBH5 ameliorated liver fibrosis and suppressed HSCs activation via triggering PTCH1 activation in an m(6)A dependent manner. Eur J Pharmacol.

[111] Zhao T, Qi J, Liu T, Wu H, Zhu Q (2022). N6-Methyladenosine modification participates in the progression of hepatitis B virus-related liver fibrosis by regulating immune cell infiltration. Front Med.

[112] Sun R, Tian X, Li Y, Zhao Y, Wang Z, Hu Y, Zhang L, Wang Y, Gao D, Zheng S, Yao J (2022). The m6A reader YTHDF3-mediated PRDX3 translation alleviates liver fibrosis. Redox Biol.

[113] Cheng W, Li M, Zhang L, Zhou C, Yu S, Peng X, Zhang W, Zhang W (2022). New roles of N6-methyladenosine methylation system regulating the occurrence of non-alcoholic fatty liver disease with N6-methyladenosine-modified MYC. Front Pharmacol.

[114] Huang K, Zhang P, Zhang Z, Youn JY, Wang C, Zhang H, Cai H (2021). Traditional Chinese Medicine (TCM) in the treatment of COVID-19 and other viral infections: efficacies and mechanisms. Pharmacol Ther.

[115] Wooton-Kee CR, Jain AK, Wagner M, Grusak MA, Finegold MJ, Lutsenko S, Moore DD (2015). Elevated copper impairs hepatic nuclear receptor function in Wilson's disease. J Clin Invest.

[116] Członkowska A, Litwin T, Dusek P, Ferenci P, Lutsenko S, Medici V, Rybakowski JK, Weiss KH, Schilsky ML (2018). Wilson disease. Nat Rev Dis Primers.

[117] Bandmann O, Weiss KH, Kaler SG (2015). Wilson's disease and other neurological copper disorders. Lancet Neurol.

